# Gene expression profiling of loss of TET2 and/or *JAK2*V617F mutant hematopoietic stem cells from mouse models of myeloproliferative neoplasms

**DOI:** 10.1016/j.gdata.2015.04.002

**Published:** 2015-04-09

**Authors:** Takuro Kameda, Kotaro Shide, Takumi Yamaji, Ayako Kamiunten, Masaaki Sekine, Tomonori Hidaka, Yoko Kubuki, Goro Sashida, Kazumasa Aoyama, Makoto Yoshimitsu, Hiroo Abe, Tadashi Miike, Hisayoshi Iwakiri, Yoshihiro Tahara, Shojiro Yamamoto, Satoru Hasuike, Kenji Nagata, Atsushi Iwama, Akira Kitanaka, Kazuya Shimoda

**Affiliations:** a Department of Gastroenterology and Hematology, Faculty of Medicine, University of Miyazaki, Miyazaki, Japan; b Department of Cellular and Molecular Medicine, Graduate School of Medicine, Chiba University, Chiba, Japan; c Division of Hematology and Immunology, Center for Chronic Viral Diseases, Graduate School of Medical and Dental Sciences, Kagoshima University, Kagoshima, Japan

**Keywords:** Microarray profiling, Loss of TET2, *JAK2*V617F, Hematopoietic stem cell, Myeloproliferative neoplasm

## Abstract

Myeloproliferative neoplasms (MPNs) are clinically characterized by the chronic overproduction of differentiated peripheral blood cells and the gradual expansion of malignant intramedullary/extramedullary hematopoiesis. In MPNs mutations in *JAK2 MPL* or *CALR* are detected mutually exclusive in more than 90% of cases [1], [2]. Mutations in them lead to the abnormal activation of JAK/STAT signaling and the autonomous growth of differentiated cells therefore they are considered as “driver” gene mutations. In addition to the above driver gene mutations mutations in epigenetic regulators such as *TET2 DNMT3A ASXL1 EZH2* or *IDH1/2* are detected in about 5%–30% of cases respectively [3]. Mutations in *TET2 DNMT3A EZH2* or *IDH1/2* commonly confer the increased self-renewal capacity on normal hematopoietic stem cells (HSCs) but they do not lead to the autonomous growth of differentiated cells and only exhibit subtle clinical phenotypes [[4], [6], [7], [8],5]. It was unclear how mutations in such epigenetic regulators influenced abnormal HSCs with driver gene mutations how they influenced the disease phenotype or whether a single driver gene mutation was sufficient for the initiation of human MPNs. Therefore we focused on *JAK2*V617F and loss of TET2—the former as a representative of driver gene mutations and the latter as a representative of mutations in epigenetic regulators—and examined the influence of single or double mutations on HSCs (Lineage^−^Sca-1^+^c-Kit^+^ cells (LSKs)) by functional analyses and microarray whole-genome expression analyses [9]. Gene expression profiling showed that the HSC fingerprint genes [10] was statistically equally enriched in TET2-knockdown-LSKs but negatively enriched in *JAK2*V617F–LSKs compared to that in wild-type-LSKs. Double-mutant-LSKs showed the same tendency as *JAK2*V617F–LSKs in terms of their HSC fingerprint genes but the expression of individual genes differed between the two groups. Among 245 HSC fingerprint genes 100 were more highly expressed in double-mutant-LSKs than in *JAK2*V617F–LSKs. These altered gene expressions might partly explain the mechanisms of initiation and progression of MPNs which was observed in the functional analyses [9]. Here we describe gene expression profiles deposited at the Gene Expression Omnibus (GEO) under the accession number GSE62302 including experimental methods and quality control analyses.

**Table:** 

Specifications	
Organism/cell line/tissue	*Mus musculus, C57BL/6, bone marrow Lineage^−^Sca-1^+^c-Kit^+^ cells (LSKs)*
Sex	*Not applicable: each fetus was genotyped for JAK2 and TET2, but was not determined by gender; for each genotype, fetal liver cells (FLs) were collected from multiple fetuses and mixed altogether, and were transplanted into lethally irradiated recipients; therefore the gender of recipients' LSKs derived from transplanted FLs was not to be determined.*
Sequencer or array type	*Agilent-028005 SurePrint G3 Mouse GE 8x60K Microarray*
Data format	*Raw and analyzed*
Experimental factors	*Genotype: Wild type vs. TET2 knock-down (TET2KD), JAK2V617F, or TET2KD and JAK2V617F (double-mutant); JAK2V617F vs. double-mutant.*
Experimental features	*Microarray gene expression profiling to identify genes that are regulated by loss of TET2 and/or JAK2V617F.*
Consent	*Animal studies were performed in accordance with the local University of Miyazaki Ethics Committee.*
Sample source location	*Not applicable*

## **Direct link to deposited data**

http://www.ncbi.nlm.nih.gov/geo/query/acc.cgi?link_type=NCBIGEO&access_num=GSE62302&acc=GSE62302

## Experimental design, materials and methods

### Mouse usage

*JAK2*V617F transgenic mice [11] and *TET2* heterozygous knockdown (*TET2*^trap/+^) mice [5] generated by gene trapping were previously reported. They were backcrossed at least 8 times onto C57BL/6 mice (B6-CD45.2). The *TET2*^trap/trap^ mice frequently died during the neonatal period on the B6-CD45.2 background; therefore, we transplanted their fetal liver (FL) cells (FLs) to generate mouse models of myeloproliferative neoplasms (MPNs). For the transplantation, we crossed male *JAK2*V617F–*TET2*^trap/+^ mice with female *TET2*^trap/+^ mice, and obtained the following 4 experimental groups of E14.5 fetuses: *JAK2*WT–*TET2*WT (wild-type (WT)), *JAK2*WT–*TET2*^trap/trap^ (TET2-knockdown (TET2KD)), *JAK2*V617F–*TET2*WT (*JAK2*V617F), and *JAK2*V617F–*TET2*^trap/trap^ (double-mutant) (Table 1). B6 mice congenic for the CD45.1 locus (B6-CD45.1) were purchased as recipients (Sankyo Laboratory Service, Tsukuba, Japan). Animal studies were performed in accordance with the local University of Miyazaki Ethics Committee.

### Generation of mouse models of MPNs

We non-competitively transplanted 1 × 10^6^ of 4 experimental groups of E14.5 FLs (WT, TET2KD, *JAK2*V617F, double-mutant) into lethally irradiated B6-CD45.1 mice [9]. Compared with the recipients transplanted with WT cells, the recipients of TET2KD cells showed normal blood cell count, no splenomegaly, comparable overall survival duration, and minimal extramedullary hematopoiesis of the lung and liver, indicating that TET2KD cells developed only a subtle clinical phenotype as MPNs. Recipients of *JAK2*V617F cells showed leukocytosis, anemia, thrombocytosis, splenomegaly, shorter survival duration, moderate extramedullary hematopoiesis, and fibrosis in bone marrow (BM) and spleen, indicating that *JAK2*V617F cells induced clinically primary myelofibrosis (PMF)-like MPNs. Double-mutant cells showed not only the phenotype of *JAK2*V617F cell recipients, but also prolonged leukocytosis, splenomegaly, and severe extramedullary hematopoiesis, with modestly shorter overall survival. These results indicated that the combination of loss of TET2 and *JAK2*V617F worsened the disease compared to single-mutant *JAK2*V617F-induced MPNs.

### RNA and cDNA preparation for microarray gene expression analyses

To identify genes regulated by loss of TET2 and/or *JAK2*V617F, we performed microarray gene expression analyses. For the cell preparation, we sacrificed the 4 experimental groups of recipients at 10–16 weeks post-transplantation, and sorted Lineage^−^Sca-1^+^c-Kit^+^ cells (LSKs) from the BM of the recipients by FACSAriaII (BD Biosciences, San Jose, USA). WT-LSKs (n = 1), TET2KD–LSKs (n = 1), *JAK2*V617F–LSKs (n = 1), and double-mutant-LSKs (n = 1) were each pooled from 5 mice (Table 1), and preserved in TRIzol reagent. RNA samples were isolated from the each pooled 2–6 × 10^4^ BM-LSKs. cDNA samples were prepared from 2–5 ng RNA samples using the Ovation Pico WTA System V2 (NuGEN, San Carlos, CA), according to the manufacturer's instructions. The NuGEN Ovation amplification methodology uses an isothermal linear amplification using DNA/RNA chimeric primers.

### cDNA labeling and hybridization

Two μg of purified and amplified cDNA was used as input into the Agilent Genomic DNA Enzymatic Labeling Kit (Agilent Technologies, Palo Alto, CA), according to the manufacturer's instructions. The Cy3-labeled cDNA was quantified and dye incorporation determined by Nanodrop ND-1000 Spectrophotometer (Thermo Fisher Scientific, Waltham, MA). For each hybridization, 1.65 μg of Cy3-labeled cDNA was hybridized at 65 °C for 17 h to an Agilent Mouse GE 8x60K Microarray (Agilent, Design ID: 028005). After washing, microarrays were scanned using an Agilent DNA microarray scanner (Agilent).

### Microarray study design

Using the 4 experimental groups of prepared cDNA samples (each, n = 1), microarray gene expression analyses were performed. Each gene expression profile of TET2KD–LSKs, *JAK2*V617F–LSKs and double-mutant-LSKs was compared to that of WT-LSKs; and a gene expression profile of double-mutant-LSKs was compared to that of *JAK2*V617F–LSKs.

### RNA and cDNA quality control analyses

**Fig. 1:**
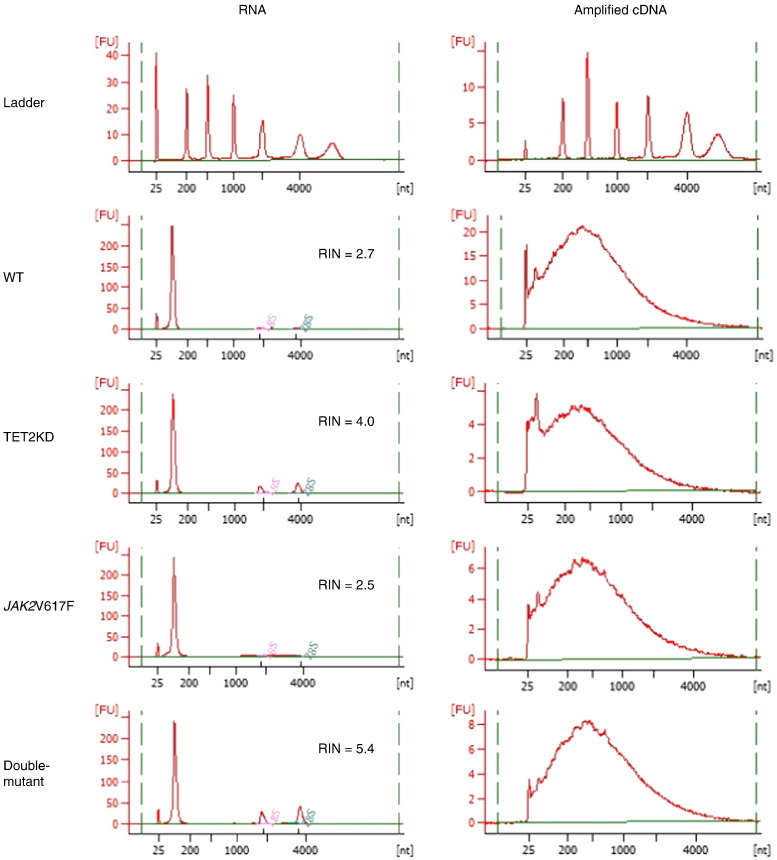
Quality control assays of RNA samples (left column) and amplified cDNA samples (right column). Bioanalyser outputs were shown for each sample. FU, fluorescence units; nt, nucleotide. RNA integrity numbers (RIN) were displayed beside each RNA plot. All amplified cDNA samples showed typical plots between 200 and 2000 kb, indicating their sufficient qualities for microarray analysis.

RNA and cDNA qualities were assessed by Nanodrop ND-1000 Spectrophotometer (Thermo Fisher Scientific) and by the 2100 Bioanalyser (Agilent). All isolated RNA samples showed appropriate A260/A280 ratios ranging between 1.56 and 1.72, but small peaks for 18 s and 28 s ribosomal RNA subunits and low RNA integrity numbers (RIN) ranging between 2.7 and 5.4 in their electropherograms, probably because of their small amounts or degradation; suggesting their insufficient qualities for the regular microarray protocol without amplification (Fig. 1 left column). However, according to the manufacturer's instructions, the NuGEN Ovation Pico WTA System V2 enables RNA samples with RIN still around 2.0 to amplify successfully and reproducibly; therefore we performed following RNA amplification and cDNA synthesis. All amplified cDNA samples showed A260/A280 ratios ranging between 1.92 and 1.96, A260/A230 ratio between 2.30 and 2.36, and typical plots between 200 and 2000 kb; indicating their sufficient qualities for the microarray protocol with amplification (Fig. 1 right column). Finally, all Cy3-labeled cDNA samples showed A260/A280 ratios ranging between 1.79 and 1.84, and dye incorporation rates ranging between 32.1 and 35.1.

### Microarray quality control analyses

**Fig. 2:**
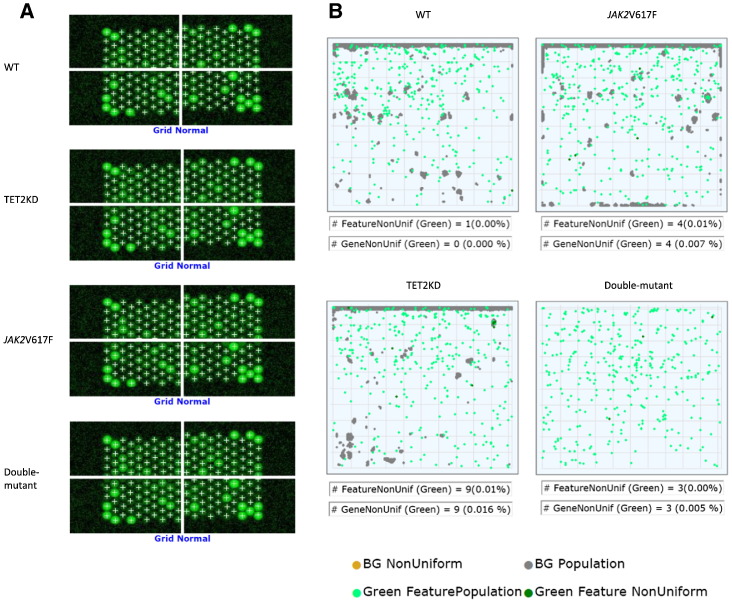
Quality control assays of hybridizations. (A) Adequate grid placements in each 4 experimental groups of hybridizations. (B) Absence of obvious biases in the distributions of outlier probes and sufficiently low frequencies (< 1%) of non-uniform features in each 4 experimental groups of hybridizations.

In each 4 experimental group of hybridization, the grid placements were adequate (Fig. 2A). Obvious biases in the distributions of outlier probes were not seen, and the frequencies of non-uniform features were sufficiently low (< 1%) (Fig. 2B).

### Data normalization

There are total of 55,681 probes on Agilent Mouse GE 8x60K Microarray (Design ID: 028005) without control probes, and intensity values of each scanned feature were quantified using Agilent Feature Extraction software version 10.7.3.1, which performs background subtractions. Normalization was performed using Agilent GeneSpring GX version 12.6.1. (per chip: normalization to 75 percentile shift).

### Data analysis (cluster analysis)

**Fig. 3:**
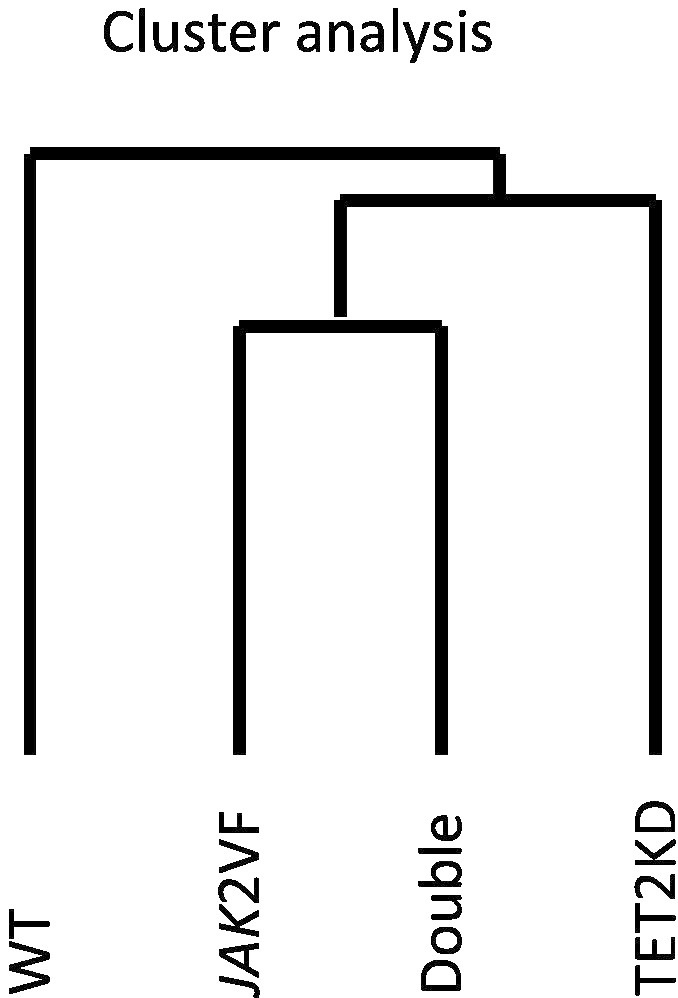
Clustering analysis of data sets from 4 experimental groups of BM-LSKs. Dendrogram constructed from unsupervised hierarchical clustering using Pearson correlation. There were close similarities in the whole-genome expression profiles between *JAK2*V617F–LSKs and double-mutant-LSKs.

Dendrogram was constructed from unsupervised hierarchical clustering of data sets from 4 experimental groups of BM-LSKs using Pearson correlation. There were close similarities in the whole-genome expression profiles between *JAK2*V617F–LSKs and double-mutant-LSKs (Fig. 3).

### Data analysis (gene set enrichment analysis (GSEA))

**Fig. 4:**
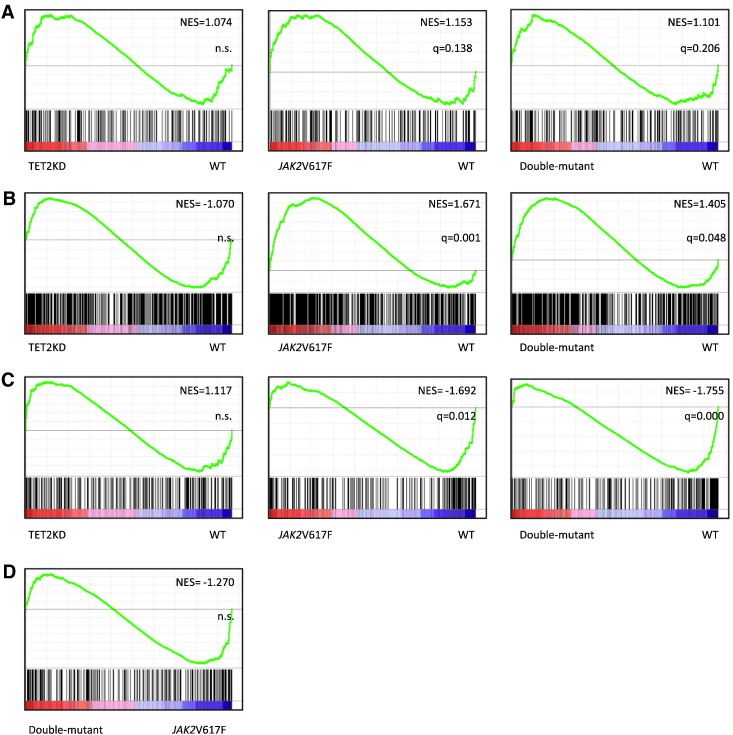
Gene set enrichment analysis (GSEA) between the expression profiles of different LSKs (WT *vs.* TET2KD, *JAK2*V617F, or double-mutant; *JAK2*V617F *vs.* double-mutant). (A) Positive enrichment of the STAT5A target genes both in *JAK2*V617F–LSKs and in double-mutant-LSKs. (B) Positive enrichment of the pre-erythroid colony-forming unit signature genes both in *JAK2*V617F–LSKs and in double-mutant-LSKs. (C) Equivalent enrichment of the HSC fingerprint genes in TET2KD–LSKs, and negative enrichment both in *JAK2*V617F–LSKs and in double-mutant-LSKs. (D) Equivalent enrichment of the HSC fingerprint genes between double-mutant-LSKs and *JAK2*V617F–LSKs. The normalized enrichment score (NES) from the overall gene expression profiles of LSKs and the false discovery rate q-value are indicated.

**Fig. 5:**
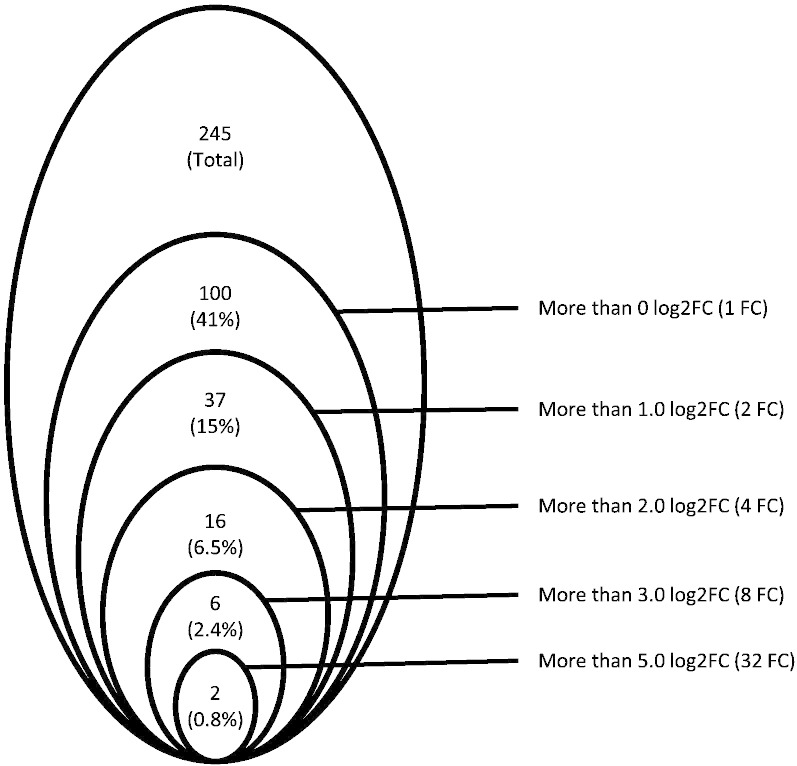
Venn diagram illustrating the numbers and frequencies of highly expressed HSC fingerprint genes in double-mutant-LSKs than in *JAK2*V617F–LSKs. Within the 245 HSC fingerprint genes, 100 (41%), 37 (15%), 16 (6.5%), 6 (2.4%) and 2 (0.8%) genes were highly expressed more than 0 log2FC (1 FC), 1.0 log2FC (2 FC), 2.0 log2FC (4 FC), 3.0 log2FC (8 FC) and 5.0 log2FC (32 FC), respectively.

Gene set enrichment analysis (GSEA) [12] was performed across the complete list of genes ranked by signal-to-noise ratio according to their differential expression. A normalized enrichment score (NES) was assigned to each gene set and the statistical significance of its enrichment was measured by FDR q-value. Consistent with the finding from cluster analysis, GSEA showed positive enrichment of the STAT5A target genes [13] and pre-erythroid colony-forming unit signature genes [14] in both *JAK2*V617F–LSKs and double-mutant LSKs, but not in TET2KD–LSKs; suggesting that *JAK2*V617F expression induced a progenitor phenotype in LSKs (Fig. 4A, B). Although there was no significant enrichment of the HSC fingerprint genes [10] in TET2KD–LSKs, it was negatively enriched in *JAK2*V617F–LSKs. Double-mutant-LSKs showed the same tendency as *JAK2*V617F–LSKs in terms of their HSC fingerprint genes, but the expression of individual genes differed between the two groups (Fig. 4C, D). Among 245 HSC fingerprint genes, 100 (41%) genes were highly expressed in double-mutant LSKs, compared to in *JAK2*V617F–LSKs; and 37 (15%), 16 (6.5%), 6 (2.4%) and 2 (0.8%) genes showed more than 1.0 log2 fold change (log2FC) (2 FC), 2.0 log2FC (4 FC), 3.0 log2FC (8 FC) and 5.0 log2FC (32 FC), respectively (Table 2, Fig. 5).

## Discussion

We present here a unique data set of mouse models of MPNs. This dataset is measured by Agilent platform and composed of gene expression profiles of normal and mutant LSKs (WT, *JAK2*V617F, TET2KD, double-mutant) (Table 1). *JAK2*V617F-induced HSC impairments were identified in several mouse models of MPNs including ours [9], [15], [16]. In our model, though single-mutant *JAK2*V617F-cells could initiate and promote MPNs during a short-term, they showed reduced self-renewal capacity *in vitro* and reduced long-term oncogenic capacity *in vivo* [9]. However, the double-mutant cells showed increased self-renewal capacity and could initiate and promote MPNs over the long-term [9], indicating that *JAK2*V617F-induced HSC impairments were restored by combined loss of TET2. Also in human, those impairments were identified in *JAK2*V617F–HSCs [17], and combined loss of TET2 seemed to restore the *JAK2*V617F-induced HSC impairments and expand the HSC compartment by altering transcriptional programs [17], [18]. Therefore, we tried to uncover the restoration mechanisms by using a gene-profiling approach in mouse models. Here, we showed that many HSC fingerprint genes were down-regulated in *JAK2*V617F–LSKs, but the expressions of significant number of them were restored in double-mutant-LSKs (Table 2, Fig. 5), though we could not see statistically significant restoration of the profile (Fig. 4D). These expressional changes might partly explain the mechanisms of initiation and progression of MPNs. In spite of our and other studies [9], [18], [19], the precise mechanisms by which loss of TET2 restores the *JAK2*V617F-induced HSC impairments still remain poorly known, further wet and dry investigations are necessary to uncover them more precisely.

## Conflict of interest

The authors declare no competing financial interests.

## Funding

This work was supported in part by Grants-in-Aid for Scientific Research (grants 24641408, 24591401, and 26860738) from the Ministry of Education, Science, Sports, and Culture in Japan.

## Figures and Tables

**Table 1:** Four experimental groups defined by the *JAK2*-and-*TET2* mutation status of the experimental tissues or cells.

Annotation	*JAK2* status	*TET2* status
WT	*JAK2WT*	*TET2WT*
TET2KD	*JAK2WT*	*TET2*^trap/trap^
*JAK2*V617F	*JAK2*V617F	*TET2WT*
Double-mutant	*JAK2*V617F	*TET2*^trap/trap^

**Table 2:** HSC fingerprint genes expressed more highly in double mutant-LSKs than in *JAK2*V617F–LSKs. For each HSC fingerprint gene, Agilent probe ID, RefSeq ID, normalized expression values (log2) in each mutant LSKs and the value of the log2 fold change (log2FC) (Double mutant *vs. JAK2*V617F) are shown. HSC fingerprint genes are arranged in descending order of the value of the log2FC.

Probe ID	RefSeq ID	Gene symbol	WT	TET2KD	*JAK2* V617F	Double-mutant	log2FC (double mutant *vs. JAK2*V617F)
A_55_P2174935	NM_001081235	MN1	0.230	− 0.936	− 5.367	− 0.130	5.237
A_51_P469480	NM_009535	YES1	− 4.917	− 4.721	− 4.618	0.502	5.120
A_55_P2078710	NM_029413	MORC4	2.775	0.403	− 4.103	− 0.382	3.721
A_52_P17098	NM_001161620	MPP7	− 2.307	− 1.290	− 2.043	1.666	3.709
A_51_P384148	NM_013649	RYK	− 5.134	− 4.928	− 5.348	− 1.843	3.505
A_55_P2121985	NM_013829	PLCB4	− 0.339	− 0.537	− 1.038	2.245	3.283
A_65_P16750	NM_001163609	PSMA8	− 4.958	− 4.351	− 4.187	− 1.193	2.994
A_51_P501803	NM_010451	HOXA2	− 3.562	− 2.678	− 5.015	− 2.069	2.946
A_51_P258409	NM_010423	HEY1	− 4.939	− 1.361	− 4.175	− 1.249	2.926
A_52_P121342	NM_020252	NRXN1	0.839	1.021	− 2.792	0.010	2.802
A_55_P2129271	NM_001285498	TEAD2	− 3.023	− 1.465	− 3.308	− 0.683	2.625
A_55_P1955457	NM_010345	GRB10	− 1.329	− 1.564	− 3.263	− 0.660	2.603
A_51_P293781	NM_016863	FKBP1B	− 4.324	− 1.063	− 3.222	− 0.629	2.593
A_55_P2066463	NM_010135	ENAH	− 5.220	− 5.021	− 4.031	− 1.655	2.376
A_52_P162486	NM_007542	BGN	0.864	1.299	− 5.224	− 2.941	2.283
A_55_P1966644	NM_013838	TRPC6	0.286	− 1.267	− 3.580	− 1.300	2.280
A_51_P462102	NM_133911	GPR125	− 4.796	− 2.431	− 2.045	− 0.073	1.972
A_66_P136228	NM_030690	RAI14	− 1.642	1.334	− 5.468	− 3.539	1.929
A_66_P134991	NM_028360	TTC19	− 3.783	0.111	− 1.617	0.297	1.914
A_55_P1981155	NM_153537	PHLDB1	− 3.791	− 2.611	− 5.050	− 3.227	1.822
A_55_P2262136	NM_013690	TEK	− 1.750	− 2.354	− 5.453	− 3.726	1.727
A_55_P2154659	NM_023651	PEX13	− 4.951	− 4.765	− 1.839	− 0.187	1.652
A_51_P258493	NM_011067	PER3	− 5.172	− 2.979	− 4.253	− 2.680	1.573
A_66_P114381	NM_001005341	YPEL2	0.865	2.139	− 2.649	− 1.103	1.547
A_55_P2163319	NM_008817	PEG3	− 5.166	− 4.958	− 5.380	− 3.871	1.510
A_55_P2036026	NM_028783	ROBO4	0.744	0.297	− 1.038	0.469	1.506
A_55_P2137887	NM_010820	MPDZ	− 5.192	− 5.020	− 5.454	− 3.969	1.485
A_55_P2000758	NM_019832	GKAP1	0.273	0.416	− 1.831	− 0.398	1.433
A_51_P350453	NM_013743	PDK4	− 3.333	− 4.200	− 5.482	− 4.071	1.410
A_51_P463003	NM_021367	TSLP	− 2.800	− 4.719	− 3.351	− 2.000	1.351
A_55_P1968362	NM_011599	TLE1	0.466	1.850	− 0.451	0.865	1.316
A_55_P2038056	NM_080285	CTTNBP2	− 4.706	− 4.534	− 5.502	− 4.272	1.230
A_51_P361220	NM_008055	FZD4	− 2.366	− 1.957	− 4.903	− 3.674	1.229
A_51_P249930	NM_146236	TCEAL1	− 5.003	− 3.211	− 3.418	− 2.247	1.171
A_52_P20048	NM_011544	TCF12	0.393	− 1.130	− 1.777	− 0.661	1.116
A_55_P2092859	NM_013471	ANXA4	2.783	1.534	− 0.644	0.459	1.103
A_55_P2109559	NM_001013616	TRIM6	− 2.055	− 3.687	− 3.141	− 2.101	1.041
